# *N*-Heterocyclic Carbene-Platinum Complexes Featuring an Anthracenyl Moiety: Anti-Cancer Activity and DNA Interaction

**DOI:** 10.3390/ijms20174198

**Published:** 2019-08-27

**Authors:** Sébastien Harlepp, Edith Chardon, Mathilde Bouché, Georges Dahm, Mounir Maaloum, Stéphane Bellemin-Laponnaz

**Affiliations:** 1 INSERM UMR_S1109, Centre de Recherche d’Immunologie et d’Hématologie, 67085 Strasbourg CEDEX, France; 2 Institut de Physique et Chimie des Matériaux de Strasbourg, Université de Strasbourg, 67034 Strasbourg, France; 3 Institut Charles Sadron, 23 rue du Loess, 67000 Strasbourg, France

**Keywords:** antitumoral activity, platinum, N-heterocyclic carbene, two-state interaction, AFM and optical tweezers microscopy

## Abstract

A platinum (II) complex stabilized by a pyridine and an N-heterocyclic carbene ligand featuring an anthracenyl moiety was prepared. The compound was fully characterized and its molecular structure was determined by single-crystal X-ray diffraction. The compound demonstrated high in vitro antiproliferative activities against cancer cell lines with IC_50_ ranging from 10 to 80 nM. The presence of the anthracenyl moiety on the N-heterocyclic carbene (NHC) Pt complex was used as a luminescent tag to probe the metal interaction with the nucleobases of the DNA through a pyridine-nucleobase ligand exchange. Such interaction of the platinum complex with DNA was corroborated by optical tweezers techniques and liquid phase atomic force microscopy (AFM). The results revealed a two-state interaction between the platinum complex and the DNA strands. This two-state behavior was quantified from the different experiments due to contour length variations. At 24 h incubation, the stretching curves revealed multiple structural breakages, and AFM imaging revealed a highly compact and dense structure of platinum complexes bridging the DNA strands.

## 1. Introduction

Inorganic compounds have an enormous impact in medicine, particularly in the treatment of cancer. Since Rosenberg’s [1,2] discovery of the cytotoxicity of cisplatin, it has found wide use as a chemotherapeutic in clinics and cisplatin continues to be used in 50–70% of all patients suffering cancer, usually administered with other drugs in combination therapy [3]. It is especially effective in treating testicular [4] and ovarian [5] carcinoma but is also used to treat other types of cancers [6]. Although the use of cisplatin in clinics has been a breakthrough in the treatment of cancer, it still faces limitations. In addition of exhibiting high systemic toxicity leading to significant side-effects [7,8] that can be minimized by dose adjustment [9,10], it is inactive against many cancer cell lines and metastases (secondary cancers).

An alternative to cisplatin could be proposed by using either other metallic compounds or by modifying the nature of the side groups. In that respect, N-heterocyclic carbenes (NHC) [11,12] have become privileged ligands in coordination chemistry [13,14] finding multiple applications ranging from homogeneous catalysis to material science [15,16,17,18,19,20,21,22,23]. More recently, these systems have also been studied in medicinal chemistry with promising successes [24,25,26,27,28]. Indeed, the use of N-heterocyclic carbene ligands offers new opportunities for biological applications in part thanks to their ease of derivatization and the high stability of their corresponding metal complexes. Apart from the antimicrobial properties demonstrated by various gold(I) [29,30], silver(I) [31,32,33,34,35,36,37], ruthenium(II), and rhodium(I) derivatives [38,39], N-heterocyclic carbene transition metal complexes have also demonstrated promising results as anticancer agents. Several NHC complexes have been studied as anticancer metallodrug candidates, especially gold [40,41,42,43], silver [44,45], palladium [46,47], and platinum [48,49], displaying antitumor activities through multiple cytotoxicity mechanisms. Among these metals, the development of platinum-based complexes bearing novel ligands is a hot topic to overcome the severe limitations encountered with the use of the world’s bestselling anticancer drug cisplatin, e.g., systemic toxicities sometimes accompanied by acquired cell-resistance mechanisms during the treatment.

**Figure 1:**
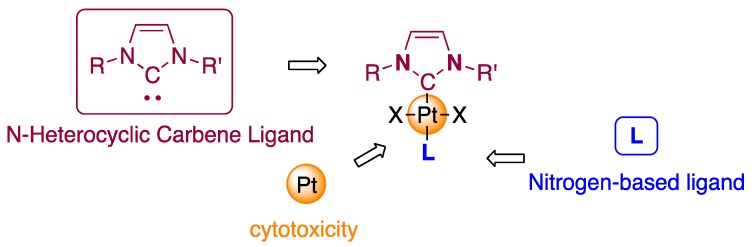
Chemical structures of platinum N-heterocyclic carbene (NHC) complexes that show high cytotoxic activities against cancer cells.

It has been demonstrated that with NHCs, encouraging results were obtained with N-heterocyclic carbene *trans*-configured complexes of general formula (NHC)PtX_2_(L) (X = halogen, L = nitrogen-containing ligand, Figure 1) [48,49]. Such complexes are highly effective as anticancer agents against various cancer cell lines, including cisplatin-resistant cells [50]. They usually exhibit cytotoxic activities with IC_50_ at a micromolar range and most of them outperform cisplatin.

Tremendous efforts have been dedicated to understand the mechanism of cytotoxicity of cisplatin [Pt(NH_3_)_2_Cl_2_] [51], and DNA has been suggested as the main intracellular target of such Pt-based antitumor agent [52]. The mechanism is proposed to involve DNA binding of the platinum center [50], causing DNA binding and cell cycle arrest, which ultimately triggers programmed cell death (apoptosis). In the case of cisplatin, the complex is hydrolyzed and the two chloride anions are exchanged by two molecules of water. When the aqueous cisplatin interacts with DNA, the water molecules are easily exchanged by the N7 atoms of guanine (or adenine) to form adducts. The cisplatin-DNA adducts are mostly intrastrand crosslinks as well as interstrand crosslinks [53]. Notably, cisplatin is also able to significantly distort the double helix toward the major groove but also to induce a partial unwinding in the proximity of adducts sites.

**Figure 2:**
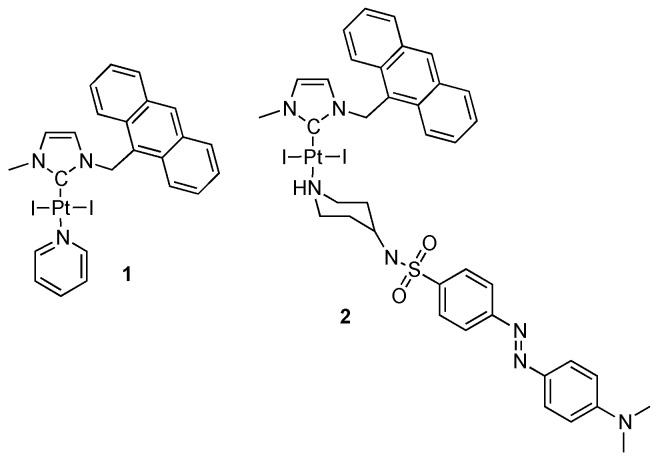
Molecular structure of the NHC platinum complexes used in this study.

On the other hand, little is known about the intracellular cytotoxic mechanism of NHC-Pt complexes mainly due to their novelty [54,55]. We thus decided to conduct several studies on the interaction between DNA and such type of complexes. For this purpose, we selected platinum complex **1** (NHC)PtI_2_(pyridine) (Figure 2), and we also used a related complex (complex **2**) to evidence the interaction of NHC-Pt complex with DNA by means of luminescence Fluorescent Resonance Energy Transfer (FRET) studies.

In this paper, we evaluated the biological activity of complex **1** and then investigated with optical tweezers and atomic force microscopy (AFM) the elastic behavior of single DNA molecules interacting with the platinum-based molecule. We show that this compound effectively decreases the contour length of DNA and induces DNA compaction. Interestingly, in comparison with cisplatin, this complex has an enhanced ability to shorten DNA at low concentrations.

## 2. Results

### 2.1. Molecular Structure of Complex ***1*** and Antiproliferative Activities

**Figure 3:**
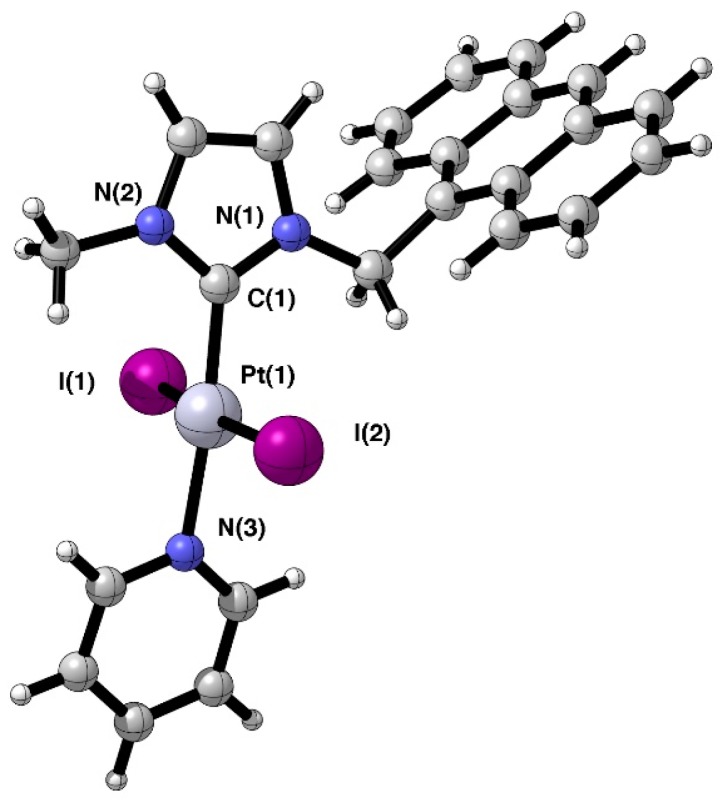
Molecular structure of the platinum complex **1**. Selected bond lengths (Å) and angles (°): C(1)-Pt(1), 1.961(1); N(3)-Pt(1), 2.088(3); Pt(1)-I(1), 2.5732(3); Pt(1)-I(2), 2.6013(3); C(1)-Pt(1)-N(3), 174.88(14); C(1)-Pt(1)-I(1); 87.72(10); C(1)-Pt(1)-I(2), 92.03(10); I(1)-Pt(1)-I(2), 172.401(14); N(1)-C(1)-Pt(1)-I(1), −88.3(3); N(2)-C(1)-Pt(1)-I(1), 87.9(3).

Suitable crystals of complex **1** for X-ray diffraction studies were obtained from CH_2_Cl_2_/*n*-hexane. Figure 3 displays the molecular structure of the compound along with selected bond and geometrical parameters. A summary of crystal and refinement data is included in supporting information. The complex displays the coordination of the pyridine ligand *trans* to the N-heterocyclic carbene ligand with the expected square-planar geometry at the platinum center. The two iodide ligands adopt the *trans* geometry [I-Pt-I angle of 172.401(14)°] and point perpendicularly toward the NHC ligand plane [N(2)-C(1)-Pt(1)-I(1), 87.9(3)] and the pyridine plane. The carbene-platinum and the platinum-N bond distances were found to be 1.961(4) Å and 2.088(3) Å, respectively, thus illustrating the strong *trans* effect of the σ-donor NHC ligand.

The in vitro antiproliferative activity on tumor cells of the NHC platinum complex **1** was evaluated by MTS colorimetric assay against a panel of 4 cancer cell lines, namely MCF7 (breast carcinoma), HCT116 (colon cancer cells), PC3 (prostate adenocarcinoma), and SKOV3 (ovarian cancer cells) along with 2 noncancer cells MRC-5 (human fetal lung fibroblast cells) and EPC (endothelial progenitor cells). IC_50_ values are summarized in Table 1 (in μM). Overall, these biological assays revealed that the platinum complex exhibits very high cytotoxic activity below the micromolar range. The IC_50_ values are ranging from 0.01 μM to 0.08 μM. The average reactivity of this compound is, therefore, of two orders of magnitude higher on average compared to cisplatin, which has been used as reference.

### 2.2. Contour Length Behavior at the Single DNA Molecule Level in Interaction with Platinum Complex ***1***

**Figure 4:**
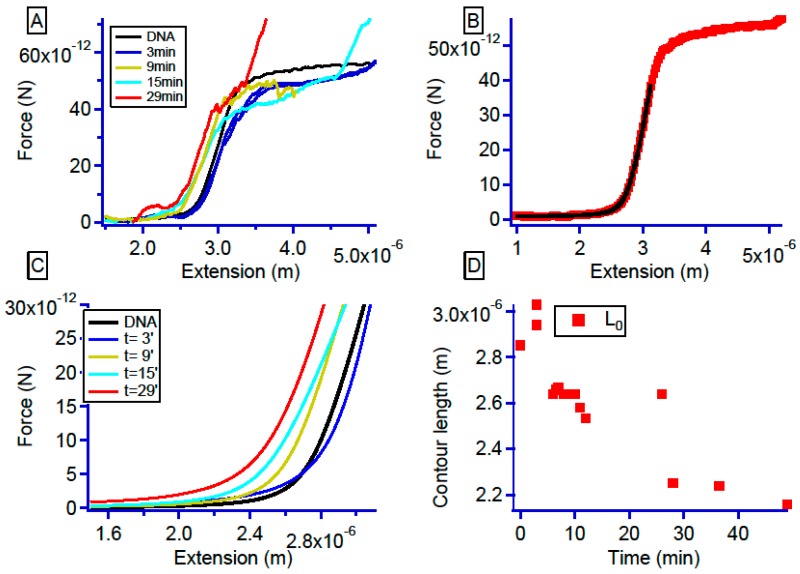
(**A**) DNA–platinum complex force-extension curves evolution at different incubation time. The curves evolve from the raw DNA (black) curve, to short time range around 3 min where DNA length increases (dark blue) before it decreases continuously over longer times (gold, light blue, and red). (**B**) Raw DNA stretching curve (red) with the modified Marko–Siggia WLC model (black). The adjusting parameters in that case are for the contour length L_0_ = 2.87 µm and for the persistence length L_p_ = 40 nm in good agreement with the tabulated values. (**C**) For more clarity, the fit obtained from the curves represented in the panel A with the same color codes. (**D**) Contour length of the DNA molecules as a function of time. The length after 3 min is 3.1 µm this length the decreases from 2.7 to 2.24 µm after 29 min of incubation.

To shed light on the molecular interaction between the platinum compound and DNA, we performed sets of single-molecule experiments. With the use of optical tweezers, mechanical behavior at the molecular level can be evidenced. These mechanical behaviors are directly linked to the molecular interaction of DNA with the added drug [56,57,58]. Before adding the platinum compound, we first checked the force-extension curve of naked DNA molecules and adjusted the resulting curves with the modified Marko–Siggia Worm-like Chain model described in Wang et al. [59]. Then, to follow the kinetics, we injected into the flow cell, a solution containing 1 µM of platinum compound (1 DNA_bp_ for 200 drug molecules) and investigated its effect on DNA. By moving the piezoelectric stage, we stretched DNA to the force-induced melting plateau and then relaxed it to its initial position (Figure 4A).

For pure DNA stretching, after adjusting the Worm Like Chain (WLC) model, we got an average length over tens of curves of 2.85 (±0.1) µm and a persistence length of 48 (± 8) nm (Figure 4B). These values are in good agreement with the expected values due to our salt conditions. After introduction of the platinum complex, we continued to perform stretching on the DNA molecule. Surprisingly, the general behavior represented in Figure 4A was not monotonous. Figure 4C shows how the mechanical response of the DNA-Pt adducts evolves over time. Past the first 5 min, the contour length of the DNA molecule increased from 2.85 µm to 3.1 (±0.2) µm. This 9% size increase was then followed by a continuous decrease in size from 3.1 to 2.64 µm (±7% of length decrease from the initial length).

**Figure 5:**
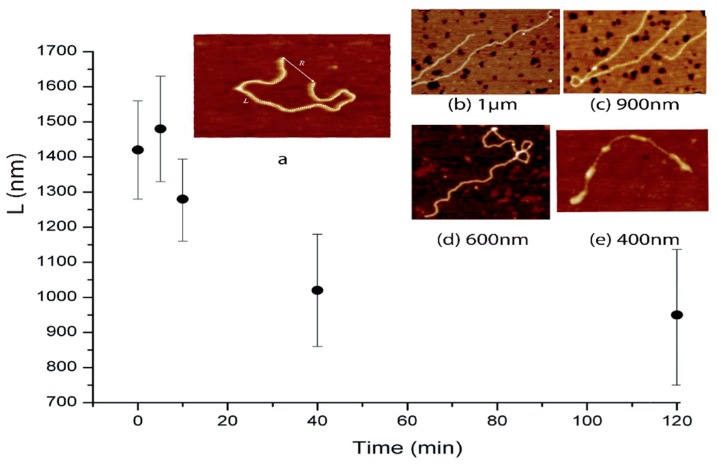
DNA length extracted from the atomic force microscopy (AFM) images as a function of platinum complex incubation time. The contour length L increases rapidly after 5 min before a continuous decrease. The image shows the DNA (**a**) conformations and length at (**b**) 5, (**c**) 10, (**d**) 40 min, and (**e**) 20 h. The sizes on bottom of the images represent the horizontal full scale of the images represented.

To confirm this result and get better insights on the observed behavior we used atomic force microscopy to visualize the general trend of the molecular transition observed with the optical tweezers. Figure 5 shows the images obtained with the AFM. From these images acquired at different time points, we extracted the contour length by summing the different segment lengths (Figure 5 panel a). The different lengths obtained over time are represented on the main graph in Figure 5. Again, one can observe at short times (around 5 min) that the contour length of the DNA increases before a decrease over time. These two methods confirm this 2-state behavior in the interaction. On the different panels (b to d), one can also observe that the number of crossings increases over time. One can hypothesize that this platinum complex induces interstrand bounds. Nevertheless, if we compare panel a (pure DNA) to panel b and panel d, not only the number of crosslink changes but also the number of kinks on the DNA strands. On panel a, the DNA molecule changes orientation with smooth angles, whereas on panel d the reorientations are abrupt with angles close to 70° on small distances.

These observations extracted from both experiments, suggest a 2-state interaction to occur. The first state consists of a length increase of the DNA molecule. Usually, this increase is attributed to intercalation of aromatic compounds into the base stacking [57]. Even if we do not have full evidence of this intercalation, we hypothesize that in our case anthracene could play this role. The second state is a condensation of the DNA molecule with an apparent shorter contour length. In the case of cisplatin, this corresponds to the intra and interstrand reactions [60]. 

Putting these 2 behaviors together (even if intercalation is of full evidence) as chemical reactions we get following:

(1) $$\{\begin{array}{c} \left[D\right]+\left[\mathrm{Pt}\right]{⇌}_{k_{2}}^{k_{1}}{\left[\mathrm{DPt}\right]}_{i}\\ {\left[\mathrm{DPt}\right]}_{i}{⇌}_{k_{4}}^{k_{3}}{\left[\mathrm{DPt}\right]}_{A} \end{array}$$

where D stands for DNA base pairs, [DPt]_i_ stands for the “intercalated” DNA and [DPt]_A_ stands for the DNA platinum condensed form.

One can write the kinetic reaction and the initial conditions for that system:

(2) {∂∂t[D]=−k1[D][Pt]+k2[DPt]i,∂∂t[DPt]i=k1[D][Pt]−(k2+k3)[DPt]i+k4[DPt]a,∂∂t[DPt]a=−k4[DPt]a+k3[DPt]i,[Pt][DNA]0=ratio,[D]0=10−9,[DPt]i0=[DPt]a0=0

We solved these equations using Wolfram Mathematica software. The kinetic parameters used to solve the equations were found from previous works and summarized in Table 2. Usually, people perform titrations and thus, only the thermodynamic constants are reported. Nevertheless, this dissociation constant is linked to the kinetic constants in the following way:

(3) $$K_{d}=\frac{k_{\mathrm{off}}}{k_{\mathrm{on}}}$$

where K_d_ stands for the dissociation constant, k_on_ for the kinetic constant in the direction of the expected reaction and k_off_ the kinetic constant in the opposite direction.

No further adjustments were operated except taking into account the relative concentrations of the different compounds used for the different experiments performed.

To obtain the relative concentrations of the different products over time, all the constants have to be multiplied by D[0], whereas the concentrations have to be divided by D[0]. D[0] corresponds to the initial DNA concentration. The measured parameter is the contour length of the DNA molecule. We have to assume a linear distribution in length of the molecule depending on the ratio of the products formed. The equation obtained with that assumption is:

(4) $$\left\{ \begin{array}{l} L_{\mathrm{DNA}}{(t)=\alpha *L}_{\mathrm{dsDNA}}{(t)+\beta *L}_{{\mathrm{DNA}}_{i}}{(t)+\gamma *L}_{{\mathrm{DNA}}_{a}}\left(t\right)\\ \alpha +\beta +\gamma =1 \end{array}\right.$$

where α, β, and γ represent the ratios of dsDNA, intercalated DNA, and complexed DNA, respectively, from the kinetic equations and L_dsDNA_ is the length of the double-stranded DNA = 0.34 nm, L_DNAi_ the intercalated length = 0.37 nm, and L_DNAa_ the complexed DNA length = 0.22 nm. These lengths were extracted from the experimental results obtained with AFM and the optical tweezers. As can be seen in Table 1, the reactivity of the molecule is around 200 times higher than the one obtained for cisplatin, taking into account this value and the relative concentrations of the product used.

### 2.3. Luminescence Studies to Understand the Underlying Interactions

**Figure 6:**
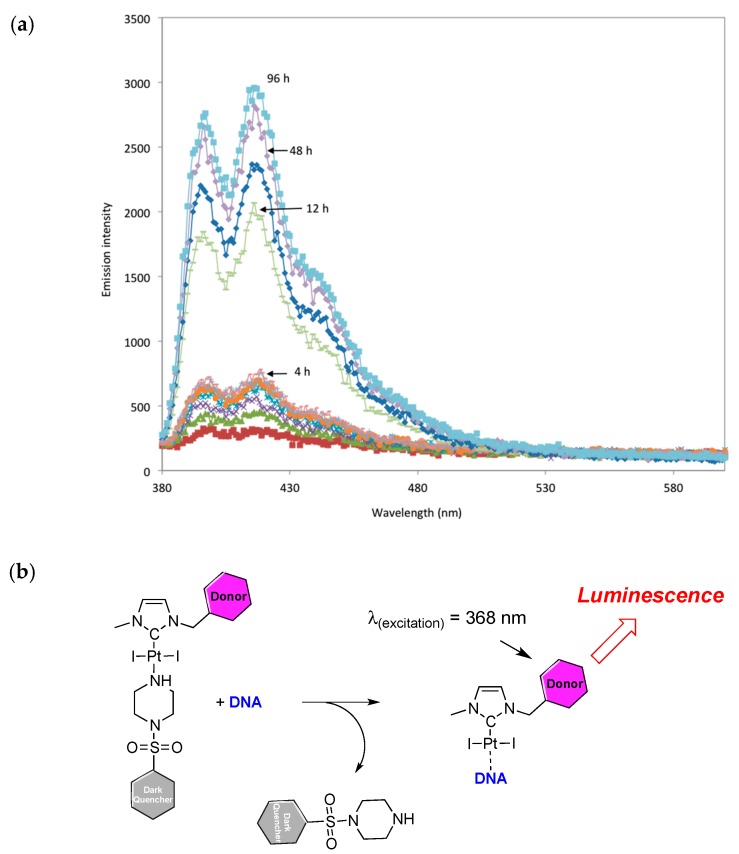
(**a**) Measurements of the emission spectra after excitation at λ = 368 nm (0.29 mg of **2** were dissolved in 50 µL of DMSO and added to a solution of 1.8 mg of salmon sperm DNA in water (1.950 mL), C_Pt_ = 1.34 × 10^−4^, (10 base pairs compared to Pt)). (**b**) Origin or the luminescence upon interaction with DNA (50).

To shed light on these two assumptions (intercalation followed by condensation), luminescent studies were performed. First of all, we have to determine whether the ligand located *trans* to the NHC carbene is labile in contact with DNA and thus allows metal–DNA interactions. To show this effect, we used molecule **2** (Figure 2), which contains a dabsyl moiety used as a ‘dark quencher’ when bound on the Pt metal [68]. After addition of dsDNA in solution, the luminescence was recorded over time. A slight evolution (Figure 6) was observed after 5 h of reaction at room temperature. Nevertheless, a significant increase of the luminescence was observed after prolonged heating at 40 °C for four days. Such result indicates a release of the dark quencher from the platinum which is most likely due to the formation of a DNA-NHC-platinum adduct. The chemical reaction describing the observed behavior is also shown in Figure 6. Nevertheless, this chemical reaction does not allow showing the release of one of the iodine ligand in order to create a compaction-reactive product as it is described in the chemical reactions described above (Equation (1)).

**Figure 7:**
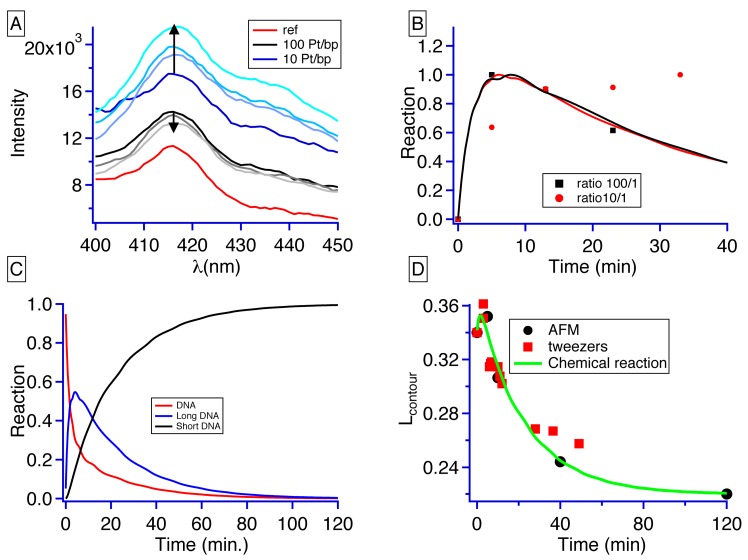
(**A**) Luminescence studies on the interaction between DNA and platinum. Two ratios were used in this study. The black curves represent the 100 Pt/bp ratio whereas the blue ones represent the 10 Pt/bp ratio. Time evolution is represented by the color gradient that varies from dark to light colors. The time schedule varies as follow darkest curve is 1 min of incubation, then 10, 20, and 30 min are represented. (**B**) The dots represent the evolution of the normalized maximal intensity obtained on each curve as a function of time. The lines are the normalized chemical model for the intercalation as a function of time and relative concentration. (**C**) Chemical evolution of the different species in solution as a function of time. (**D**) Evolution of the contour length as a function of time obtained with the AFM measurements (black dots), the optical tweezers (red squares) and the simulation green line.

To show that our compound can intercalate into the DNA base pairs, luminescent studies were performed on complex **1** in interaction with DNA. First, the platinum complex is excited at 360 nm, and luminescence is acquired from 400 to 450 nm (red curve on Figure 7A). Later the experiments are performed with a mix of DNA and this compound at 2 different ratios over time. The two ratios are 100 platinum complexes (black curves in Figure 7A) per DNA base pair and 10 platinum complexes over DNA base pairs (blue curves in Figure 7A). The first curve was recorded around 3 min after the addition of the platinum compounds and all other curves at a ten minutes later time point.

Two different behaviors are observed. As for the ratio of 10, the luminescence increases over time (from dark blue to light blue) while the ratio of 100 continuously decreases over time (from black to light grey) (Figure 7A). From these raw luminescent curves, we took the maximal value, and for each ratio normalized these values so that the overall behavior varies from 0 to 1 (Figure 7B). The theoretical curves obtained from the resolution of the system Equation (6) were superimposed for both cases. The only parameter that was changed in these curves was the ratio. The theoretical curves adjust the experimental data points and in the case of the intercalation this model can be used.

This model allows the monitoring of all the different compounds over time (Figure 7C). For a ratio of 200, after 5 min, DNA is totally consumed in its native form and the intercalated structure (long DNA) dominates. Around 20 min, there is as much intercalated DNA than the adduct one. At 50 min, the reaction reaches its steady state, the adduct DNA represents 90% of the total structure whereas the intercalated one represents around 10% of the global structure. Introducing this result in Equation (7), we obtain an average change in the contour length represented on the green curve in Figure 7D. On the same curve, we represented the contour length obtained from the AFM experiment and the optical tweezers experiment. The average behavior of the theoretical curve mimics nicely the experimental data points.

### 2.4. Persistence Length Behavior

**Figure 8:**
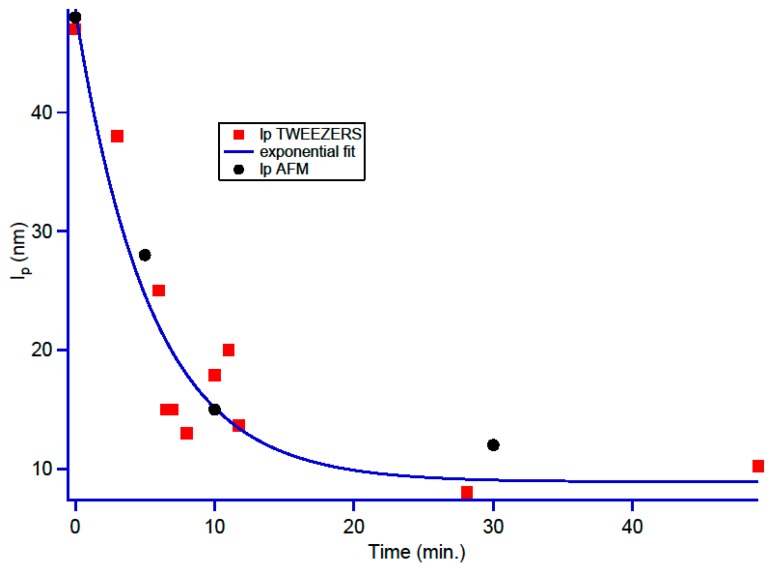
Persistence length decrease as a function of time for the optical tweezers (red dots) and AFM (black dots). An exponential adjustment was added on top of the experimental points to help visualization.

From the traction curves, one can also extract a second parameter, namely the persistence length. From the AFM images it is also feasible as described in Mantelli et al. [69], using Equation (4). The comparison of these persistence lengths obtained from both experiments is summarized in Figure 8.

The persistence length decreases continuously from 48 nm (native DNA in buffer condition) to 10 nm after 30 min of incubation with the platinum compound. For easier visualization of the overall scheme, we adjusted these results with an exponential decrease.

The persistence length decrease has been described in the case of the cisplatin [66,67,70,71] and 10 nm lengths have already been measured in cisplatin compounds [49]. Taking into account the different models established for cisplatin the decrease in the persistence length can be attributed either to the formation of monoadducts that locally changes the DNA skeleton structure or to the formation of diadducts. These diadducts have two possibilities to act on the persistence length either by increasing the number of kinks when intrastrand adducts are formed or by forming micro-loops due to interstrand adducts [66,67,70,71]. Nevertheless, Li et al. [71] have compared the persistence lengths of DNA incubated either with transplatin or cisplatin and conclude that the formation of diadducts between the molecule and the DNA strand is necessary to observe persistence lengths as low as the one obtained with **2** Nevertheless, it takes long time for complex **2** to interact with DNA and to form diadducts, which is why the effects are recorded after 20 h.

### 2.5. Long Time Incubation Behavior

**Figure 9:**
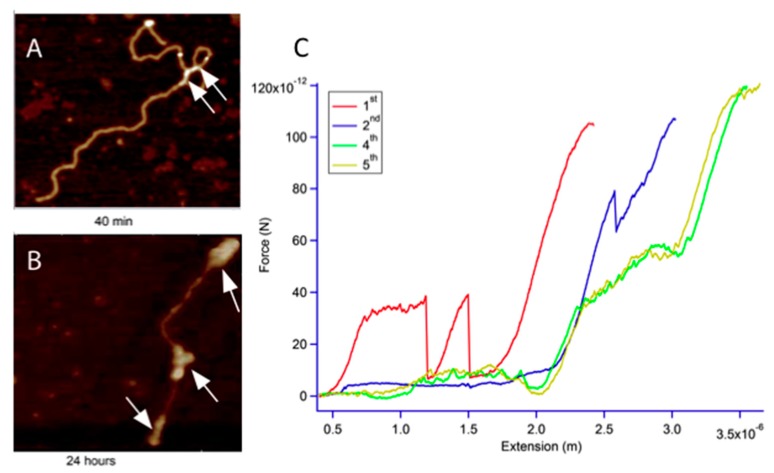
(**A**) DNA molecule incubated 40 min with the platinum compound. Interstrand crosslinks appear after 40 min (arrow). (**B**) DNA molecule incubated 24 h with the platinum compound. Compact structures appear (white arrows). (**C**) Successive force-extension curves on the same DNA molecule after 24 h incubation with the platinum compounds.

According to the literature, diadducts can be interstrand and, in this case, DNA should show kinks in its structure. These kinks are clearly seen on the AFM images after 40 min of incubation (Figure 9A), but they can also be interstrand. In that case, DNA should form loops with different sizes, which is observed in this case after 40 min. This reaction is quite slow and upon 20 h incubation, as in Figure 9B, aggregates are visible on the DNA chain.

Stretching on these molecules after 20 h incubation gives rise to a curve that differs from the typical curves presented in Figure 9C. The various curves represent a series of successive force-extension curves obtained on the same molecule. The first curve shows an increase in force after only 0.5 µm of extension and reaches a pseudo-plateau at a force starting at 32 pN and ending at 38 pN over an extension of 600 nm. At this extension, an unexpected force drop appears. The force sinks from 38 pN to 8 pN rapidly and corresponds to a win in length which equals 90 nm. The force then increases again following the WLC model till the force reaches 38 pN and a similar drop is observed. Following stretching, the molecule behaves normally except that the molecule does not undergo a B-S transition even at forces as high as 100 pN. The second stretching curve goes further in extension which seems to be due to some losses in interaction between the platinum complex and the DNA strand. This curve does not present any transition but around 80 pN a drop appears. This drop is around 23 pN and occurs at a force that is around twice the force needed in previous experiment. Further stretching gave superimposable curves, the full contour length of the DNA molecules was not reached, and a B-S transition appeared. This B-S transition is shorter as the 1.7 full extensions which are expected and the transition is not as cooperative as in naked DNA. This can be seen by the transition forces that normally have a slope close to zero and ∆F is around 5 pN. In our case, in presence of platinum complexes, the transition starts around 30 pN and ends around 60 pN, which gives ∆F = 30 pN. This behavior has been described for cisplatin by Krautbauer et al. [56]. They reported a ∆F value around 40 pN and the transition was starting at 30 pN and ending at 70 pN. The 5th stretching shows a second transition around 120 pN which again was described by Krautbauer et al. This second transition was attributed to the melting of the strands.

## 3. Discussion

NHC-platinum complex **1** which features an anthracene moiety has been designed with the aim of increasing the overall cytotoxic activity of the system because of potential synergistic effect. The compound was easily synthesized in good yield in one step and was found to be highly stable both in solid state and in solution (DMSO/water). The molecular structure has been unambiguously established by X-ray diffraction studies. Antiproliferative activity of **1** was measured (IC_50_, Table 1) on a panel of five different human cancer cells (MCF7, HCT116, PC3, SK-OV3, and MRC-5) and one noncancerous cell line EPC, using cisplatin as a reference for the study. The compound showed very high activity with IC_50_ ranging from 0.01 to 0.08 µM, which are two orders of magnitude higher than cisplatin activity. This is also much higher than known platinum(II) N-heterocyclic carbene complexes thus confirming the positive impact of the anthracene moiety. Indeed, complexes of type (NHC)PtI_2_(L) have IC_50_ at a micromolar range typically from 0.5 to 5 µM [48,54,55,68,72].

It is widely recognized that the anticancer activity of cisplatin and related compounds such as oxaliplatin is a consequence of DNA damage further triggering cell death. In vitro DNA binding studies have pointed out the role of such interactions to induce apoptosis in such family of NHC-platinum complexes [73]. In order to gain more insight into the nature of the metal–DNA interaction, a dye pair was used for FRET spectroscopy (complex **2**, Figure 2). The anthracene fragment on the backbone of the NHC has been used as a donor fluorophore which efficiently transfers its energy to the dabsyl moiety, thus quenching the anthracene emission. The emission may be recovered upon the release of the dabsyl ligand from the platinum complex thus evidencing the lability of the pyridine ligand in the *trans* position [68]. Herein, we investigated the luminescence properties of complex **2** in the presence of salmon sperm DNA. The luminescence is visible after few hours and the relative intensity is gradually increasing as a function of time. Overall, these results are consistent with a pyridine-DNA ligand exchange thus leading to the formation of a platinum–DNA adduct upon release of the free pyridine.

To further investigate the interaction of this platinum NHC complex with DNA, single-molecule experiments were performed using either optical tweezers or AFM microscopy in aqueous environment. Both experiments showed clear interaction between the strands and the platinum complex. Analyzing the contour length in both experiments strikingly revealed a two-state behavior during the experiments. At shorter time scales, between 0 and 7 min, the contour length significantly increased by 6% (from 0.34 nm/bp to 0.36 nm/bp). At longer time scales, between 8 to 120 min, and even longer (24 h), the contour length monotonously decreased from 0.34 nm/bp to 0.22 nm/bp. Anthracene complexes are known to intercalate the base pairs (64), thus we hypothesize this interaction to initiate the inter and intrastrand interactions occurring in both cisplatin and transplatin at longer time scales [64]. The duality in the interactions based on the chemical structure of the compounds, as well as the lability of the pyridine ligand, is in good agreement with a two-state interaction. A monotonous decrease is also observed over time of the persistence length of the DNA molecule from 50 nm to 12 nm, which is in good agreement with measurements established on cisplatin/DNA complexes [65]. The decrease in persistence length brings the DNA molecule to be more flexible and thus to favor short- and long-distance interactions which, over time, brings the DNA/NHC complex to form compact and dense molecules. This compaction over 12 h of interaction has been described at the single molecule level in the cisplatin compounds [62]. We performed single-molecule stretching on these dense compounds to characterize the forces involved in this densification. We measured a force of around 40 pN to break part of the interactions and to further be able to stretch the DNA molecule. The successive stretching on the same DNA strand revealed that once these adducts broke, they were not able to form again at the time scale used to perform the successive stretching. This study conducted at the molecular and cellular level shows a strong affinity of these new NHC compounds to interact strongly with the DNA strands and to induce cellular apoptosis at doses that are around 100 times lower than the classical platinum compounds administered in clinics. Although gaps are evident between this molecular study and the in vitro observations and the in vivo effects, it appears that these new NHC complexes have higher cellular toxicity than cisplatin which is commonly used in clinics.

## 4. Material and Methods

### 4.1. NHC-Pt Complexes: Synthesis and Characterization

NHC platinum complex **1** has been synthesized from the corresponding imidazolium as previously reported [68,72]. The platinum complexes were prepared by reaction of the imidazolium salt in the presence of platinum dichloride, excess NaI and K_2_CO_3_ in dry pyridine at 100 °C overnight, under nitrogen atmosphere. The compound was purified by silica chromatography and was recrystallized to get the expected product in 63% yield. The molecular structure of the Pt complex was unambiguously confirmed by X-ray crystallography. The synthesis of the NHC complex **2** has been previously reported by us [68].

### 4.2. DNA and Optical Tweezers

For the force-extension measurements, we prepared unconstrained DNA molecules functionalized on one end with the biotin and digoxygenin at the other end to allow the attachment to streptavidin-coated beads and the anti-digoxygenin-coated flow cell, respectively [74].

The optical setup and the way to stretch DNA were previously described in different works [74,75]. This DNA construct consists of a construct of a dimer of commercially available plasmid DNA PBR322 (Sigma Aldrich, St. Louis, MO, USA) with a total size of 9.7 kbp and a length of 2.8 µm [74]. We incubated 1 ng of this DNA which represents a concentration of reactive base pairs of 5 nM. The single-beam trap obtained through a high numerical aperture objective catches the dielectric particle linked to DNA. The other side of the DNA molecule was attached to the coverglass which is moved in a controlled way with a piezoelectric translation stage. All the stretching experiments were performed at a pulling rate of ~500 nm/s in 10 mM Hepes.

The obtained force-extension curves were post-analyzed using the modified worm-like chain model described first by Bustamante et al. in 1994 [76].

(5) $$F=\left(\frac{k_{B}T}{L_{p}}\right)\left[\frac{1}{{4(1-x/L}_{0}{+F/k}_{0}{)}^{2}}-\frac{1}{4}+\frac{x}{L_{0}}-\frac{F}{k_{0}}\right]$$

where L_p_ represents the persistence length, L_0_ the contour length, k_0_ the elastic modulus, and k_B_T the thermal energy.

Another elegant method to follow the lengths changes was described by Baumann et al. [77]. By taking into account only the entropic part of the obtained curve, but for extension x approaching L_0_, the worm-like chain equation can be rewritten as follows:

(6) xL0=1−12(kTFLp)12

Or expressed differently, we get a linear relation between the inverse square root of the force and the applied extension:

(7) F−12=(1−xL0)(4LpkT)12

### 4.3. DNA and AFM

The DNA used in these experiments was obtained from an EcoRI linearized plasmid PBR322 (Sigma Aldrich, St. Louis, MO, USA). This plasmid is a 4361 bp long DNA that corresponds to a contour length of 1480 nm. DNA was purified after every step using chromatography columns from Qiagen (QIAquick PCR Purification Kit, Venlo, The Netherlands). All DNA preparations were diluted in 1 mM tris–HCl buffer, pH 7, to a final DNA concentration of 1 µg·mL^−1^.

To perform experiments on naked DNA, MgCl_2_ added to the DNA solution to a final concentration of 1 µM. 200 µL of this DNA solution was injected in AFM liquid cell (Brucker, Billerica, MA, USA) and DNA molecules adsorbed onto freshly cleaved mica at room temperature. In the case of the solution containing platinum complex, no MgCl_2_ was added. DNA and compound **2** were incubated at room temperature for the time decided for the experiment before being injected in the AFM cell following the same procedure as detailed above.

Images were collected using a Nanoscope IIIa (Veeco Inc, New York, NY, US) operated in tapping mode in solution. Ultrasharp, noncontact silicon cantilevers (NanoAndMore, Wetzlar, Germany) with a nominal tip radius of <10 nm were driven at oscillation frequencies in the range of 20–26 kHz. During AFM imaging, the force was reduced to avoid dragging of DNA by the tip. The scan rate was usually 1.4 Hz. Integral gain was adjusted to give sharp images. Images were taken without online filtering and were subsequently processed only by flattening to remove the background slope. Apparent DNA contour lengths were measured by summing the consecutive distances between the starting and ending positions that are displayed on the image. From AFM images of DNA molecules, we measured end-to-end distance R and the apparent DNA lengths L_0_.

The rigidity of DNA is often characterized by its persistence length L_p_. It provides a measure of the distance along which the direction of the chain persists before changing its course. Herein, we describe a method for measuring the persistence length of dsDNA in physiological conditions. During adsorption, DNA molecules are transformed from three dimensional (3D) into two dimensional (2D) objects. Two extreme cases were reported: one where the molecules freely equilibrate on the surface, before being trapped in a particular conformation; and one where the molecules adhere without having equilibrated on the substrate, resembling the natural 3D conformation. In the latter case, the conformation of the molecules reflects the history of their approach to the surface and it is, therefore, difficult to distinguish between the intrinsic conformation and those induced upon adsorption. Recently, we have shown that in the case of DNA adsorption in solution, the molecules freely equilibrate on the surface [69]. In this case, it is possible to obtain an ensemble of 2D conformations, which can be related to the true 3D DNA structure as:

(8) $${\langle R^{2}\rangle }_{2D}{=4L}_{p}L\left(1-\frac{{2L}_{p}}{L}\left({1-e}^{-\frac{L}{{2L}_{p}}}\right)\right)$$

where <R^2^> is the mean square end to end distance of the chain in two dimensions and L is the contour length. For long chains <R^2^>_2D_ = 4L_p_ × L.

### 4.4. Luminescence Studies on the Complexes

Upon excitation of **2** at 368 nm, the fluorophore’s emission is quenched by the dabsyl group (i.e., *dark quencher* of the excitation energy of the anthracene moiety; dabsyl = dimethylaminoazobenzenesulfonyl). The luminescent properties of the anthracene moiety may be recovered upon a release of the dabsyl quencher from the platinum complex (i.e., ligand exchange of the dark quencher *trans* to the carbene). A solution of **2**, dissolved in DMSO, was added to a solution of salmon sperm DNA in water (10 DNA base pairs compared to platinum, C_Pt_ = 1.34 × 10^−4^ M).

A solution of **1**, dissolved in DMSO, was added to a solution of salmon sperm DNA in water (1 DNA base pairs compared to 10 or 100 platinum, C_Pt_ = 4 × 10^−7^ M). The luminescence is expected to increase as the complex intercalates between the DNA base pairs (excitation at 368 nm).

### 4.5. Evaluation of Cell Viability by MTS Assays

Samples were prepared by dissolution of the compound in DMSO and dissolution of cisplatin in water) at stock concentrations of 10 mM. MCF7, HCT116, PC3, and SK-OV3 cell lines were maintained as monolayers in RPMI 1640 medium supplemented with 10% fetal calf serum, in the presence of penicillin, streptomycin, and fungizone in 75 cm^2^ flask under 5% CO_2_, while MRC5 and EPC were grown in complete D-MEM medium. Cells were plated in 96-well tissue culture plates in 200 μL complete medium at a density of 1000–2500 cells per well and treated 24 h later with 2 μL of compounds using a Biomek 3000 automation workstation (Beckman-Coulter, Pasadena, CA, USA). Controls received the same volume of the appropriate vehicle (DMSO or water, 1% final volume). After 72 h exposure, MTS reagent (CellTiter 96^®^ Aqueous One, Promega, Madison, WI, USA) was added and incubated for 3 h at 37 °C; the absorbance was monitored at 490 nm and results expressed as the inhibition of cell proliferation calculated as the ratio [(1-(OD490 treated/OD490 control)) × 100] in triplicate experiments after subtraction of the blank without cells. Positive controls (cells incubated with a reference drug at its IC_50_ concentration) were routinely added to check the responsiveness of cells. For IC_50_ determination (50% inhibition of cell proliferation), cells were incubated for 72 h following the same protocol with compound concentrations ranged 5 nM to 100 μM in separate duplicate experiments. At these concentrations, no interference with Pt complexes was noticed at 490 nm.

## 5. Conclusions

In conclusion, we have shown that a platinum diiodo complex stabilized by a pyridine and an N-heterocyclic carbene ligand featuring an anthracenyl moiety has high in vitro antiproliferative activities against several cancer cell lines, with IC_50_ values below 100 nM. The anthracenyl moiety has been used as a luminescent tag to gain more insights into the metal–DNA interactions. We also performed single-cell experiments using either optical tweezers or AFM microscopy, and cisplatin was used as a control since its interaction with DNA has been widely studied. We confirmed the ability of the platinum complex to interact with the DNA strands and revealed a highly compact and dense structure of platinum compounds bridging the DNA strands. Overall, our investigations confirmed that the platinum NHC complex behaves similarly to cisplatin.

## Figures and Tables

**Table 1:** In vitro cytotoxic activity of platinum complex **1** against several cell lines (after 72 h of incubation; stock solutions in DMSO for complex **1**; stock solution in H_2_O for cisplatin); values in µM.

Compound	MCF7	HCT116	PC3	SK-OV3	MRC-5	EPC
1	0.08 ± 0.01	0.01 ± 0.01	0.03 ± 0.01	0.02 ± 0.01	0.02 ± 0.01	0.08 ± 0.01
Cisplatin	>10	3.3 ± 0.1	5.1 ± 0.4	4.4 ± 0.8	8.5 ± 0.1	2.2 ± 0.1
Reactivity Ratio	>125	330	170	220	425	27.5

MCF7, breast carcinoma. HCT-116, colon cancer cells. PC3, prostate adenocarcinoma. SKOV3, ovarian cancer cells. MRC-5, human fetus cell line. EPC, endothelial progenitor cells.

**Table 2:** Different kinetic constants values used in the simulation, values from the literature.

Name	Value Literature	Reference
K_d_ = 8 µM	0.35 < K_d_ < 26 µM	[61,62,63]
k_1_ = 0.025 M^−1^·min^−1^		[60,64]
k_2_ = 2 × 10^−7^ min^−1^	K_d_ corresponds	
k_3_ = 0.055 M^−1^·min^−1^	57 < k < 8 ×10^−3^ M^−1^·min^−1^	[65]
k_4_ = 5 × 10^−9^ min^−1^	Adjusted from experiment	
K_d_ = 2.57 × 10^−8^ M	K_d_ = 10^−8^ M	[66,67]

## Supplementary Materials

Supplementary File 1
